# Case report: Important considerations for a renal mass on a solitary kidney in an adult with history of childhood wilms tumor

**DOI:** 10.3389/fonc.2022.971341

**Published:** 2022-08-03

**Authors:** Benjamin N. Schmeusser, Arnold R. Palacios, Eric Midenberg, Reza Nabavizadeh, Viraj A. Master, Shreyas S. Joshi

**Affiliations:** Department of Urology, Emory University School of Medicine, Atlanta, GA, United States

**Keywords:** Wilms tumor, solitary kidney, partial nephrectomy, secondary malignant neoplasm (SMN), ureter abnormalities

## Abstract

Adult survivors of childhood Wilms tumor are at an increased risk of secondary malignant neoplasms. The presence of a solitary kidney further complicates clinical management in this population. Herein, we present the case of a 37 year old female with a history of childhood Wilms tumor presenting with a secondary renal neoplasm. We highlight important clinical considerations for renal function preservation and present a finding of predisposition to kidney stone formation due to urinary stasis from distorted ureter architecture secondary to tumor mass effect.

## Introduction

Adults with a history of childhood Wilms tumor (WT) and chemoradiation are at an increased risk of secondary malignant neoplasms (1–4). Termuhlen et al., in their study looking at 25-year outcomes of childhood Wilms tumor survivors in the Childhood Cancer Survivor Study, reported an increased incidence of soft tissue sarcomas, adenocarcinomas, lymphomas/leukemias, and other malignant neoplasms in this population (2). Renal Cell Carcinoma (RCC) has also been observed in adults with a history of WT (1, 4). Here we report a case involving a patient with a history nephrectomy for childhood WT presenting with a new solitary kidney mass.The clinical evaluation, diagnostic process, and surgical management are first discussed. Additionally, distortion of ureter architecture secondary to tumor mass effect refractory to treatment and resulting long-term management considerations is also presented.

## Case description

A 37 year old female was referred to the urology clinic for evaluation of a renal mass in the setting of a left solitary kidney. Past medical history is significant for a right Wilms tumor (WT) status post nephrectomy at three years of age, followed by adjuvant chemotherapy and radiation for two years. The patient states the contralateral kidney was not included in her radiation. History regarding treatment and management of this patient’s childhood WT is otherwise limited by time and access to records. However, the patient was able to recall that routine monitoring revealed no signs of recurrence, radiotherapy associated complications (i.e.soft tissue dystrophy), or evidence of ureteral anomalies. Additional surgical history includes two remote cesarean sections. She initially presented with 2 weeks of abdominal pain and pressure, nausea, and constipation. Review of systems was negative for hematuria, vomiting, diarrhea, and constitutional symptoms such as fever, fatigue, night sweats, or weight loss. Physical exam revealed a palpable mass in the left upper abdomen but was otherwise soft and nontender. Creatinine (Cr) was 0.7mg/dL, eGFR=120mL/min/m^2^, and hemoglobin was 10.1g/dL. Laboratory workup was otherwise non-significant.

**Figure 1:**
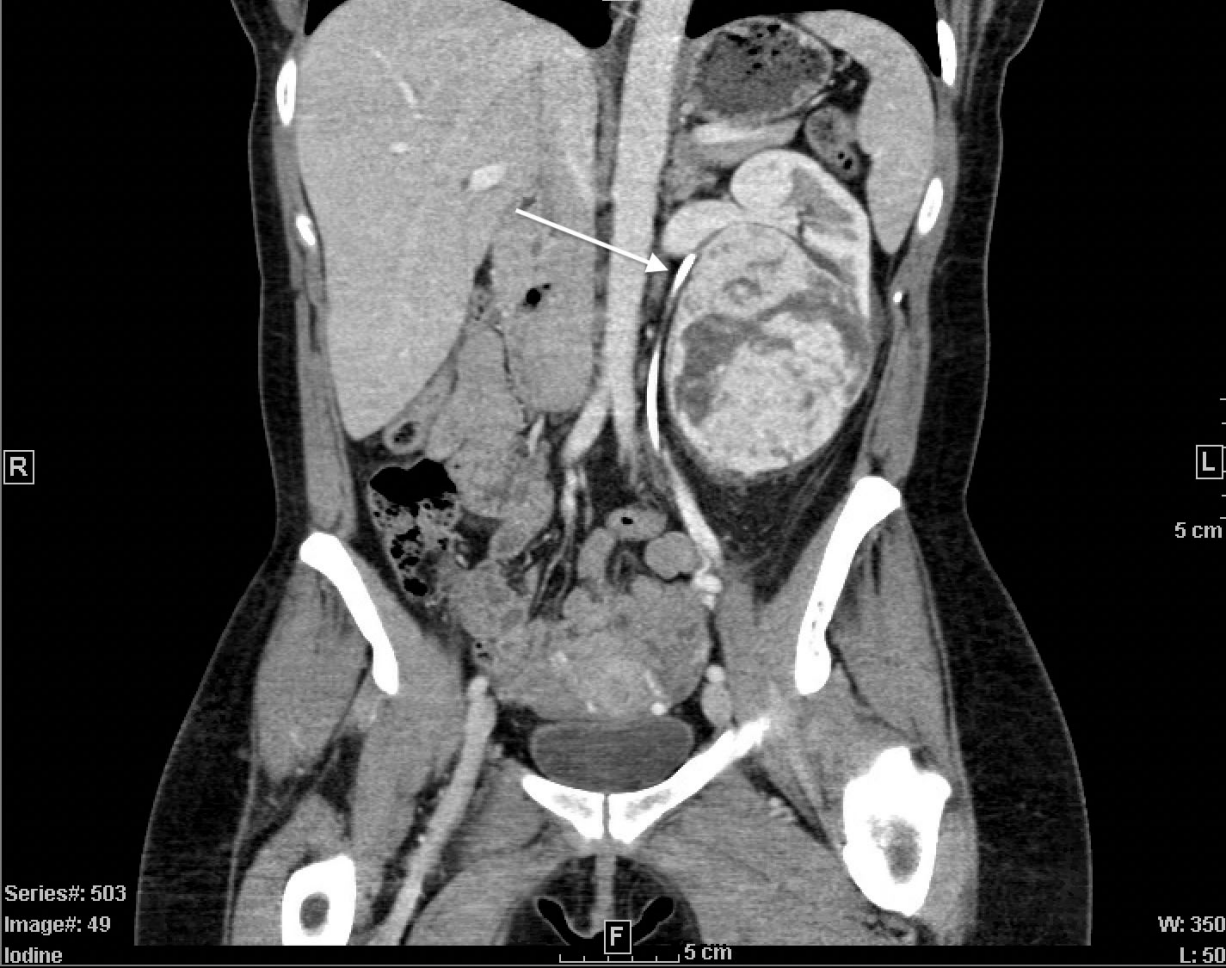
CT-Abdomen with IV contrast. Coronal view demonstrating large, 7.5x8.3x10.0cm heterogeneous solid mass in solitary left kidney. Stent in place *(white arrow)*, indicating compressed and arched ureter superior to the mass.

A CT-abdomen revealed a 7.5 x 8.3 x 10.0cm heterogeneous solid mass of the left solitary kidney with compression on the renal hilum and resulting hydronephrosis (**Figure 1**). Additionally, a 1.2cm stone was seen in the anterior lower pole. Subsequent imaging of the brain and chest revealed no evidence of metastatic disease.

**Figure 2:**
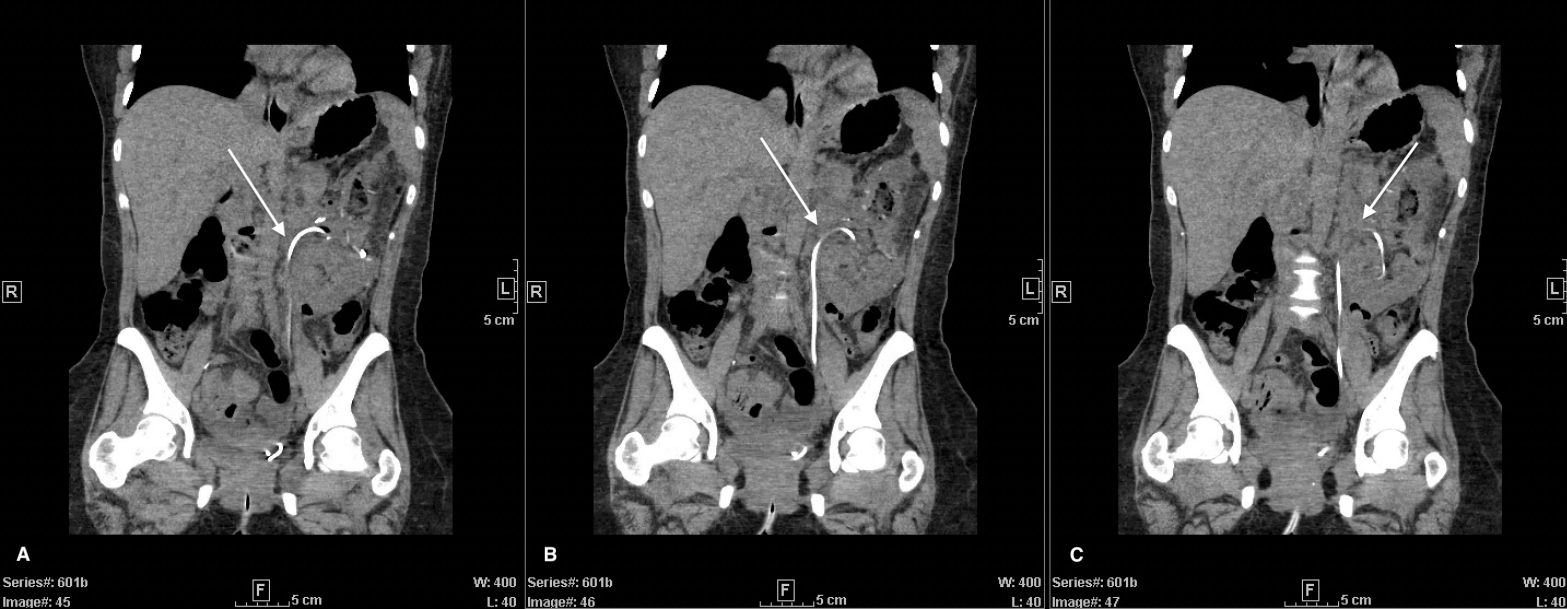
Post left partial nephrectomy CT-abdomen with IV contrast. Coronal view of 3 continuous images from left to right **(A–C)**. Ureteral stent *(white arrows)* displaying continued arching trajectory superiorly despite removal of mass.

Following tumor board consensus, a retroperitoneal percutaneous core needle biopsy of the mass was obtained. Biopsy pathology revealed an oncocytic neoplasm, indicating likely benign oncocytoma versus chromophobe renal cell carcinoma (RCC). Given unlikely improvement with neoadjuvant therapy, a complex open partial nephrectomy was planned. Nephrology was consulted to assist with perioperative management given the patient’s history of unilateral nephrectomy. Intraoperatively, after achieving adequate exposure, a bulldog clamp was placed across the renal artery and the kidney was surrounded by ice slush. Following ten minutes of cold ischemia, tumor enucleation began; however, the bulldog clamp did not appear to sufficiently control the patient’s renal arterial supply. It was decided to instead place a Satinsky clamp across the renal vein and artery, leading to a bloodless field. The enucleation was completed, and the collecting system was entered in several places which was oversewn with 4-0 vicryl sutures. Importantly, the kidney was surrounded by ice during the excision and reconstruction with a total clamp time of 120 minutes. Following clamp removal, a few venous bleeders were controlled with 5-0 monofilament suture. No significant arterial bleeding was visualized. Hemostatic materials were placed into the defect, and renorrhaphy was completed with sliding V-loc barbed sutures in standard fashion. Omentum from the transverse colon and stomach was harvested, pediculated off the left gastroepiploic artery, and tunneled into the left gutter to fill the defect. A 19Fr blake drain was placed in the left lower abdomen. A 16Fr foley catheter and ureteral stent were additionally placed. Prior to closure, retroperitoneal ultrasound revealed good renal blood flow. Total operation time was 270 minutes. Estimated blood loss was 500mL and no transfusion was required. On post-op day 3, the patient’s drain was removed and she was discharged home with a foley catheter and ureteral stent in place. The foley catheter was removed two-weeks post-discharge, and the stent was removed one-month postoperatively. Final pathology of the mass confirmed oncocytoma. Postoperative imaging revealed continued arching of the ureter in the absence of the mass (**Figure 2**). The patient’s renal function remained stable (Cr=0.7mg/mL, eGFR=107mL/min/m^2^) without need for dialysis.

**Figure 3:**
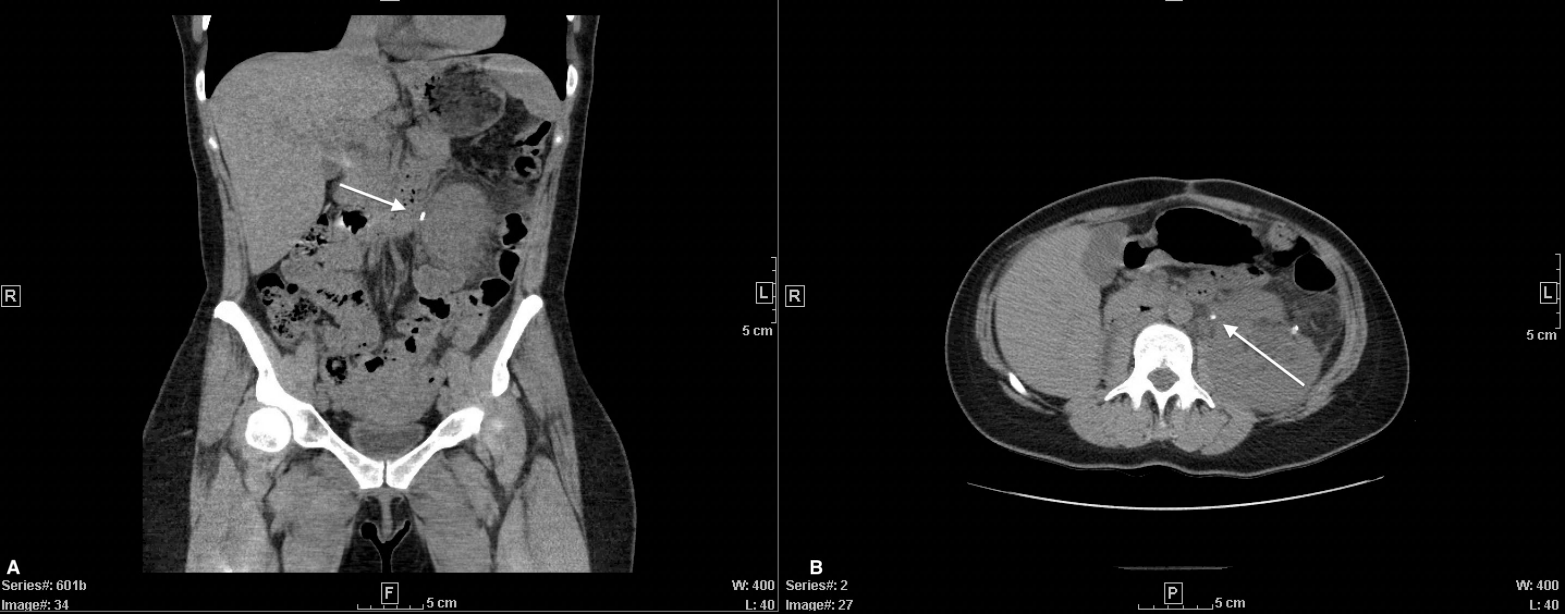
CT-Abdomen without contrast. Coronal **(A)** and axial **(B)** views displaying left-sided obstructing UPJ stone.

Short-term recovery was uncomplicated. Nine months after the surgery, she presented with nausea and left flank pain without fevers, chills, hematuria, or dysuria. Her Cr was increased to 1.4 mg/dL from 0.7 mg/dL. There was no leukocytosis and the urinalysis was benign. CT-abdomen revealed multiple left sided nonobstructing stones and a 5mm obstructing stone at the ureteropelvic junction (UPJ) with hydronephrosis (**Figure 3**). Laser lithotripsy was completed without complication. Stone composition was 90% uric acid. Intraoperative retrograde pyelogram revealed a distorted, superior malrotation of the left ureter. The patient’s propensity for urinary stasis secondary to her superiorly arched ureter is the likely cause of her stone formation. To monitor, manage, and prevent further stone development, the patient was scheduled to follow-up with endourology where she was encouraged to maintain hydration to express at least 2 liters of urine per day.

## Discussion

Renal masses in solitary kidneys are complex cases that require multidisciplinary care. As an absolute indication for nephron sparing interventions, the simultaneous optimization of renal function and oncologic control becomes crucial for maximizing patient outcomes (5, 6). In this particular case, a patient with history of childhood WT presenting with a new renal mass of the solitary kidney and subsequent anatomic distortion of the ureter secondary to mass effect, further complicates clinical management. In the following discussion, lessons learned and important considerations in this case are explored.

WT, or nephroblastoma, is the most common renal malignancy in childhood (7). The pathogenesis of childhood WT is closely tied to gene mutations and resulting disruptions in embryologic nephrogenesis (7). Most often unilateral, WT can be bilateral in up to 5-9% of cases (8). Treatment for WT is usually nephrectomy with adjuvant chemotherapy and radiation, as in our case, though neoadjuvant chemotherapy to monitor response can be considered (7). Survival rates for WT have been reported to be around 90%, though more aggressive histology can be associated with only 50% survival (8). Recurrence, associated with only a 50% survival rate, occurs in 20% of patients, typically within the first 2 years (8). Although extremely rare, delayed WT recurrence, defined as 5 years after initial diagnosis, have been reported in the literature (9, 10). Secondary renal neoplasms arising in adults with history of childhood WT have also been reported and include ccRCC and other RCC subtypes, oncocytomas, metanephric adenomas, and atypical cysts (1, 4).

Renal masses in young adults (17-45 years old), have been demonstrated to be benign in up to 20% of cases (11, 12). However, given the patient’s history of WT and chemotherapy/radiation, which increases her risk for a secondary malignant renal neoplasm, a preoperative percutaneous biopsy of the mass was done to prevent unnecessary surgery and best direct treatment (1, 4). The result of the biopsy in this case was oncocytic neoplasm. Although reassuring that WT was not the cause and negating the need for systemic therapy, an oncocytic neoplasm on the pathology report fails to differentiate between a benign renal oncocytoma and chromophobe RCC (13). Chromophobe RCC, though a lower grade and more favorable subtype of RCC, is still recommended to be treated with nephrectomy for optimal oncologic control (14, 15).

Due to the potential for malignancy and symptoms of mass effect, partial nephrectomy was selected as the treatment choice. However, partial nephrectomy in solitary kidney patients presents additional long-term postoperative challenges and considerations for kidney function due to absence of contralateral compensation. A preoperative nephrology consult is recommended in these cases for optimal management and in preparation for worst-case scenarios, such as renal replacement therapy given the anatomic complexity inherent in this case (16). Predictors of better renal recovery postoperatively include younger age, higher preoperative eGFR, and greater renal parenchyma preservation (16, 17). Although ischemia time–both cold and warm–during partial nephrectomy has long been proposed as negative predictor of postoperative eGFR, studies suggest it has a minimal contribution with renal parenchymal preservation and preoperative eGFR having a more significant effect (16–19). Ching et al. examined patients that underwent partial nephrectomy in a solitary kidney at 5 and 10 years postoperatively, and found that patients who eventually required permanent dialysis or transplantation had a median eGFR of 26.1mL/min/1.73m^2^ compared to 46.7mL/min/1.73m^2^ for patients not requiring such interventions (17). Nevertheless, following partial nephrectomy in a solitary kidney, eGFR is expected to decline immediately postoperatively but then plateau around one month afterwards and remain relatively stable for at least 10 years (16, 17, 20, 21).

Interestingly, in this case, the patient had continued distortion and arching of the ureter at the UPJ despite removing the mass. The superior arching path of the ureter has resulted in urinary stasis, which predisposes her to form kidney stones. The patient had no past evidence of malrotation or history of nephrolithiasis. No similar reports have been found in the literature. The patient now has an endourologist managing this anomaly. Active surveillance of these patients is essential for maximizing renal function, and ultimately minimizing long term morbidity and mortality.

## Conclusions

This case illustrates a patient with a solitary kidney secondary to nephrectomy for childhood Wilms tumor presenting with a large renal mass. Important considerations on the diagnosis and management in these patients must be made to ensure preservation of renal function and adequate oncologic control.

## Data availability statement

The original contributions presented in the study are included in the article/supplementary material. Further inquiries can be directed to the corresponding author.

## Ethics statement

Written informed consent was obtained from the individual(s) for the publication of any potentially identifiable images or data included in this article.

## Author contributions

BS and SJ conceived and designed the study. SJ, VM, and RN played a critical role in the patient’s care and decision making, as outlined in the case report. All authors contributed to the writing, editing, and revision of the manuscript. All authors approved of the submitted version.

## Conflict of interest

The authors declare that the research was conducted in the absence of any commercial or financial relationships that could be construed as a potential conflict of interest.

## Publisher’s note

All claims expressed in this article are solely those of the authors and do not necessarily represent those of their affiliated organizations, or those of the publisher, the editors and the reviewers. Any product that may be evaluated in this article, or claim that may be made by its manufacturer, is not guaranteed or endorsed by the publisher.
